# Bilateral Nipple Leiomyoma

**DOI:** 10.1155/2013/475215

**Published:** 2013-05-09

**Authors:** Ugur Deveci, Mahmut Sertan Kapakli, Fatih Altintoprak, Mine Cayırcı, Manuk Norayk Manukyan, Abut Kebudi

**Affiliations:** ^1^Maltepe University School of Medicine, General Surgery Department, 34843 Istanbul, Turkey; ^2^Sakarya University School of Medicine, General Surgery Department, 54100 Sakarya, Turkey; ^3^System Medical Laboratories, Pathology Clinic, 34940 Istanbul, Turkey

## Abstract

Cutaneous leiomyomas are benign smooth muscle neoplasms of the skin. They arise from vascular, arrector pili, genital, and areolar smooth muscles. The most common localizations of cutaneous leiomyomas are the extensor surfaces of the extremities and the trunk. To our knowledge, only few cases of one-sided nipple leiomyomas have been reported, but two-sided nipple leiomyomas have not been presented. For the first time, here, we report a bilateral nipple leiomyoma.

## 1. Introduction

Cutaneous leiomyomas are benign neoplasms of smooth muscles that sometimes occur in a multiple fashion. There are five types of cutaneous leiomyomas: (a) multiple piloleiomyomas, (b) solitary piloleiomyoma, (c) solitary genital leiomyoma, (d) solitary angioleiomyoma, and (e) leiomyomas with additional mesenchymal elements [1]. The genital group includes masses arising from the dartos muscle of the scrotum or from the labia majora, as well as those derived from the erectile smooth muscle cell of the nipple in either sex [2]. We report the case of a patient with a leiomyoma arising from bilateral nipples who presented with persistent nipples pain and tenderness. The clinical characteristics, gross and microscopic pathologic findings, and management of this lesion are discussed. 

## 2. Case Presentation

A 31-years-old female patient admitted to our clinic with a 6-month history of enlarging hard lesions on her bilateral nipple. She complained from intermittent pain in the bilateral nipple but denied nipple discharge or tactile or emotional stimuli. She did not have any other disease. The patient had no family history of breast cancer. No history of important or hereditary disease in the family was reported. She had two grown up children whom she had suckled in infancy. At no time had there been any discharge from both nipples or the ulceration of the skin. 

**Figure 1:**
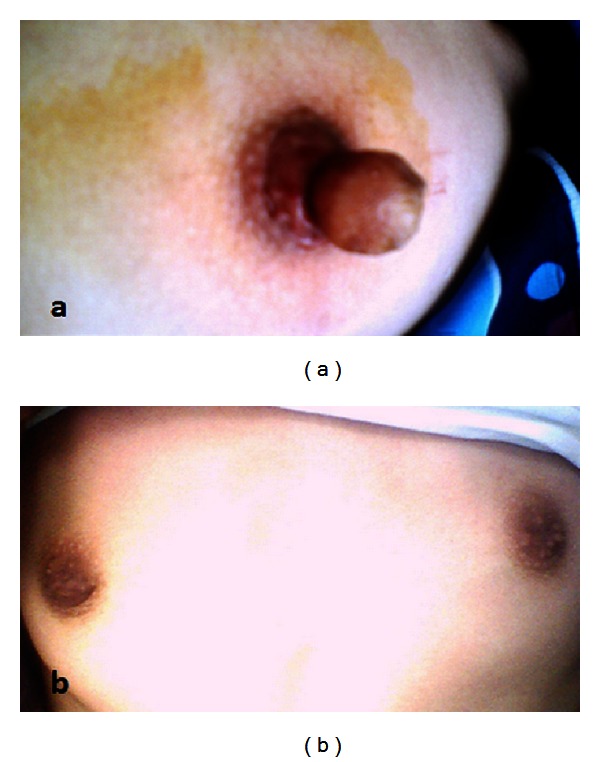
(a) The appearance before excision. (b) The appearance six months after excision.

On physical examination, there are 15 × 10 mm skin covering hard masses embedded in both nipples. There is no color change or eczematous reaction on the areola around both nipples (Figure 1(a)). The tumors extended into the pedicle which attached it to the areola. Physical examination of both breasts revealed no obvious calcification, skin distortion, inflammation, or nipple discharge. There were no palpable lymph nodes in both axillas or supraclavicular regions. During menstrual periods, no change in the size of the tumors was observed. There was a pain due to physical contact. No ultrasound abnormalities were found in the breast tissue bilaterally. The lesions were slightly hyperechoic compared with breast parenchyma. Color Doppler sonography showed no definite evidence of increased flow. Mammographic examination was not performed due to young age of patient. Laboratory examinations were normal. Ultrasonographic evaluation of the abdomen was normal. Gastroscopic and colonoscopic evaluations proved no pathologic findings.

**Figure 2:**
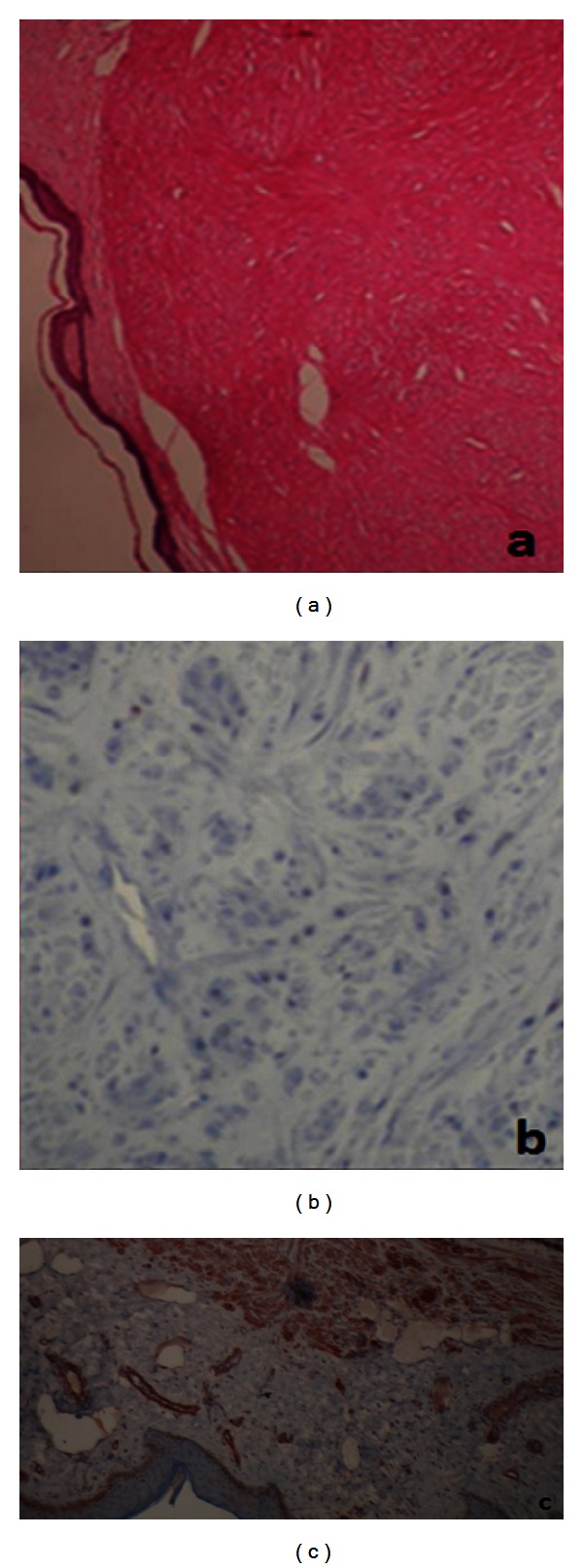
(a) H&E staining ×40. (b) Masson trichrome staining ×400. (c) SMA staining ×400.

The lesions were completely excised by local anesthesia. Frozen section study showed clear margin and no malignancy. Hematoxylin-eosin and Masson trichrome staining revealed smooth muscle fiber bundles interlace with variable amounts of collagen (Figures 2(a) and 2(b)). Immunohistochemically, positive staining by smooth muscle actin (SMA) demonstrated leiomyoma (Figure 2(c)). Six months later, bilateral breast appearance is seen in Figure 1(b). No recurrence was seen three years after the excision.

## 3. Discussion

Leiomyomas are benign soft-tissue neoplasms that arise from smooth muscle. They can develop wherever smooth muscle is present. Leiomyomas may be categorized into the following five types: multiple piloleiomyomas, solitary piloleiomyoma, angioleiomyoma (solitary), genital leiomyoma (solitary), and leiomyomas with additional mesenchymal elements [1]. Leiomyomas have been reported predominantly in adult females, with an approximate sex ratio (F : M) of 3 : 1 [3]. A family history or genetic linkage has not been reported for solitary leiomyomas. Hereditary multiple cutaneous leiomyomatosis is a tumor predisposition syndrome characterized by multiple cutaneous and uterine leiomyomas and an increased risk of developing renal cancer [4]. Multiple cutaneous and uterine leiomyomatosis, also known as Reed's syndrome, is an autosomal dominant genetic condition [5]. Uterine leiomyomas occur in more than 90% of females in this syndrome and frequently require a hysterectomy before the age of 30 years. Renal cancer ratio is 1%–17% in this syndrome. Our patient had no uterine myoma, renal mass, and family history. 

Genital leiomyoma is the least commonly occurring type of cutaneous leiomyoma, and data to establish its various epidemiological tendencies are currently inadequate [6]. The groin and nipple lesions are generally solitary asymptomatic masses, in contrast to their cutaneous counterparts, which are sometimes painful, either spontaneously or in response to cold, tactile, and emotional stimuli. The pain is thought to occur secondary to calcium-dependent contraction of smooth muscle cells within the tumor [7]. Nipple leiomyomas tend to be smaller than 2 cm in diameter and must be clinically differentiated from angiolipomas, glomus tumors, eccrine spiradenomas, neurofibromas, nevi, lipomas, Paget's disease, and breast carcinomas [8]. 

Tissue examination is necessary to establish the diagnosis. An incisional biopsy or a complete excision is indicated for tissue examination and pathologic diagnosis. Given the clinical presentation, we opted for a complete excision after discussion with the patient. Special stains can be used to distinguish smooth muscle from collagen, both of which are pinkish red on hematoxylin and eosin staining. Piloleiomyomas are smooth muscle tumors and are generally well differentiated. They occur mainly in the reticular dermis and are not encapsulated. The smooth muscle bundles of these tumors are interlaced with variable amounts of collagen. The Masson trichrome stain highlights smooth muscle as dark red and collagen as bluish-green. With aniline blue stains, smooth muscle becomes red and collagen blue. A von Gieson stain results in yellow smooth muscle contrasted against red collagen. With phosphotungstic acid hematoxylin (PTAH) stain myofibrils are purple. Immunohistochemical staining for desmin, S100, and smooth muscle actin (SMA), markers of smooth muscle differentiation, can be performed to detect these markers in piloleiomyomas [1]. 

Medical therapy plays a limited role, but calcium-channel blockers and *α*-adrenergic blockers may help in palliating or eliminating associated pain through inhibition of smooth muscle contraction [6]. Gabapentin, a novel anticonvulsant with an uncertain mechanism of action, has also been used to control piloleiomyoma-related pain [9]. The usual management of leiomyoma is complete excision. In rare cases, relapse has been reported when excision was not complete at the first surgery [10]. It must be ascertained at the time of frozen section examination that all margins are clear of residual tumors to prevent recurrence. No data in the literature exist regarding the recurrence of solitary leiomyoma after complete excision. There is no documented tendency toward malignant degeneration in cutaneous leiomyomas. 

Cutaneous leiomyomas are benign, smooth muscle tumors that may be a sign of underlying systemic disease. Increased awareness of the connection between cutaneous lesions and renal malignancy can lead to life-saving early detection. If multiple cutaneous leiomyomas are present, family history should be investigated and abdominal imaging should be performed for malignancy. The standard treatment of solitary lesions is complete surgical excision. Frozen section examination is a must to be sure for clear margin. Reexcision should be performed in the presence of close or positive margin. Complete excision is diagnostic and therapeutic.
